# Inhibition of Elevated Ras-MAPK Signaling Normalizes Enhanced Motor Learning and Excessive Clustered Dendritic Spine Stabilization in the MECP2-Duplication Syndrome Mouse Model of Autism

**DOI:** 10.1523/ENEURO.0056-21.2021

**Published:** 2021-07-03

**Authors:** Ryan Thomas Ash, Shelly Alexandra Buffington, Jiyoung Park, Bernhard Suter, Mauro Costa-Mattioli, Huda Yaya Zoghbi, Stelios Manolis Smirnakis

**Affiliations:** 1 Department of Psychiatry and Behavioral Sciences, Stanford University, CA 94305; 2 Department of Neuroscience, Baylor College of Medicine, Houston, TX 77030; 3 Medical Scientist Training Program, Baylor College of Medicine, Houston, TX 77030; 4 Department of Neurology, Brigham and Women’s Hospital and Jamaica Plain Veterans Administration Hospital, Harvard Medical School, Boston, MA 02115; 5 Memory and Brain Research Center, Baylor College of Medicine, Houston, TX 77030; 6 Department of Neuroscience, Cell Biology, and Anatomy, University of Texas Medical Branch, Galveston, TX 77555; 7 Department of Pediatrics, Texas Children’s Hospital and Baylor College of Medicine, Houston, TX 77030; 8 Department of Molecular and Human Genetics, Baylor College of Medicine, Houston, TX 77030; 9 Jan and Dan Duncan Neurological Research Institute at Texas Children’s Hospital, Houston, TX 77030; 10 Howard Hughes Medical Institute, Baylor College of Medicine, Houston, TX 77030

**Keywords:** autism, dendritic spine, ERK, MAPK, MECP2, spine clustering

## Abstract

The inflexible repetitive behaviors and “insistence on sameness” seen in autism imply a defect in neural processes controlling the balance between stability and plasticity of synaptic connections in the brain. It has been proposed that abnormalities in the Ras-ERK/MAPK pathway, a key plasticity-related cell signaling pathway known to drive consolidation of clustered synaptic connections, underlie altered learning phenotypes in autism. However, a link between altered Ras-ERK signaling and clustered dendritic spine plasticity has yet to be explored in an autism animal model *in vivo*. The formation and stabilization of dendritic spine clusters is abnormally increased in the MECP2-duplication syndrome mouse model of syndromic autism, suggesting that ERK signaling may be increased. Here, we show that the Ras-ERK pathway is indeed hyperactive following motor training in MECP2-duplication mouse motor cortex. Pharmacological inhibition of ERK signaling normalizes the excessive clustered spine stabilization and enhanced motor learning behavior in MECP2-duplication mice. We conclude that hyperactive ERK signaling may contribute to abnormal clustered dendritic spine consolidation and motor learning in this model of syndromic autism.

## Visual Abstract

**Figure:**
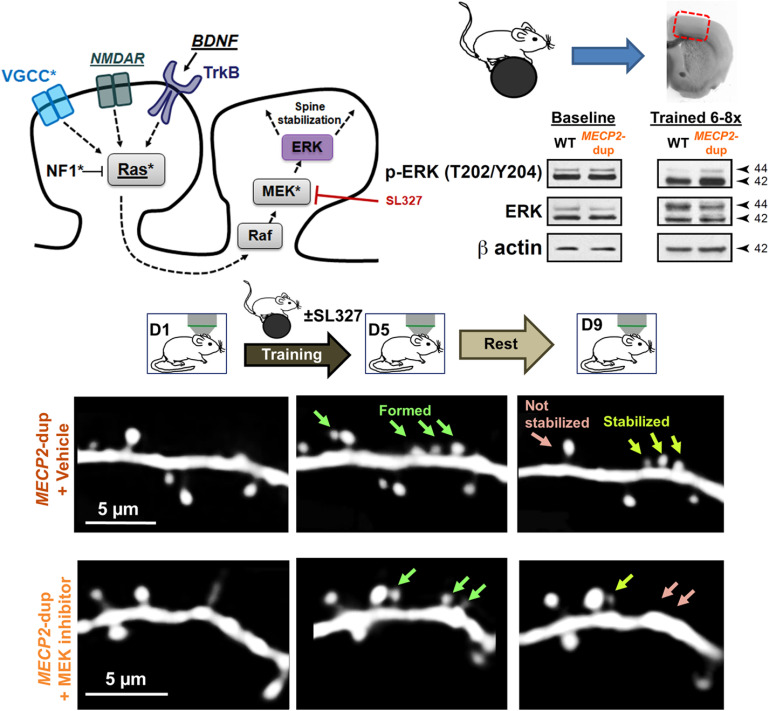

## Significance Statement

It has been proposed that autism-associated genetic mutations lead to altered learning phenotypes by perturbing cell signaling pathways that regulate synaptic plasticity in the brain. The Ras-ERK/MAPK signaling pathway, which promotes stabilization of dendritic spine clusters, has been particularly implicated in autism spectrum disorder (ASD). Here, we show that Ras-ERK signaling is increased in motor cortex following rotarod training in the *MECP2*-duplication syndrome mouse model of autism, and that the abnormal motor learning and excessive stabilization of clustered dendritic spines previously observed in MECP2-duplication mice can be rescued by pharmacological inhibition of Ras-ERK signaling. This provides additional support to hypotheses that autistic phenotypes arise from disrupted Ras-ERK signaling and synaptic plasticity and suggest potential future paths for therapeutic intervention.

## Introduction

It has been proposed that phenotypes of autism spectrum disorder (ASD) arise from an abnormal imbalance between the stability and plasticity of synaptic connections in the brain (Ramocki and Zoghbi, 2008). Syndromic autism refers to a subgroup of ASD caused by genetic abnormalities that cause autism at high penetrance (Sztainberg and Zoghbi, 2016). Modeling these genetic abnormalities in mice has led to the development of autism animal models with improved validity (Sztainberg and Zoghbi, 2016). Abnormal synaptic plasticity is a common feature in animal models of autism (Bourgeron, 2015). Some autism mouse models exhibit impaired synaptic plasticity whereas others show enhanced synaptic plasticity (Bourgeron, 2015). Understanding the molecular mechanisms underlying these plasticity changes could open new avenues for therapeutic intervention. Animal models for the methyl-CpG-binding-protein 2 (MeCP2) disorders Rett syndrome (MeCP2 loss-of-function) and *MECP2*-duplication syndrome (MeCP2 gain-of-function) in particular have led to a wealth of findings on the molecular biological and neural circuit underpinnings of ASD (Collins et al., 2004; Jiang et al., 2013; Lyst and Bird, 2015; Lu et al., 2016; Sztainberg and Zoghbi, 2016) and identified potential avenues for treatment (Na et al., 2014; Hao et al., 2015; Sztainberg et al., 2015; Achilly et al., 2021).

Enhanced repetitive motor learning on the rotarod is observed in several autism mouse models with prominent behavioral inflexibility, including *MECP2*-duplication (Collins et al., 2004; Sztainberg et al., 2015), neuroligin-3 (Rothwell et al., 2014), 15q duplication (Nakatani et al., 2009), PTEN (Kwon et al., 2006), and CNTNAP2 mice (Peñagarikano et al., 2011), providing a robust model behavior for studying the abnormal consolidation of repetitive motor routines. It was previously reported that an aberrant increase in the formation and stabilization of dendritic spine clusters in the animal model of *MECP2*-duplication syndrome correlated with an enhancement in motor performance on the rotarod task (Ash et al., 2021). The increased clustered-spine stability in the ∼9- to 10-μm proximity range that was observed is strikingly similar to the range of a known BDNF-TrkB-Ras-ERK-dependent form of clustered-spine plasticity (Harvey and Svoboda, 2007; Harvey et al., 2008; Makino and Malinow, 2011; Niculescu et al., 2018). Ras-ERK/MAPK signaling in hippocampal pyramidal neurons has been shown *in vitro* to be involved in the cooperative potentiation of neighboring dendritic spines (Harvey et al., 2008; Patterson et al., 2010; Kwon and Sabatini, 2011). When a spine is activated, Ras enters its GTP-bound active state and diffuses 9–10 μm down the dendrite to invade neighboring spines. There it initiates the ERK phosphorylation cascade orchestrating a number of transcriptional and translational changes associated with synaptic consolidation (Ye and Carew, 2010).

Mutations in Ras-MAPK pathway genes are linked to several forms of autism (Stornetta and Zhu, 2011; Wen et al., 2016; Vithayathil et al., 2018), and both patients and animal models of autism demonstrate abnormal Ras-MAPK signaling (Ebert and Greenberg, 2013; Cheng et al., 2017; Rosina et al., 2019). Ras-MAPK genes have also been shown to be dysregulated in *MECP2*-duplication mice (Chahrour et al., 2008). We therefore hypothesized that hyperactive ERK signaling could contribute to the abnormal plasticity phenotypes observed in our animals.

Here, we show that Ras-ERK signaling is upregulated in the motor cortex of *MECP2*-duplication animals after motor training, and both excessive synaptic clustering and enhanced motor learning can be reversed by pharmacological normalization of ERK signaling. These data link a structural dendritic spine phenotype to a specific autism-associated cell-signaling pathway in a mouse model of autism, *in vivo*.

## Materials and Methods

### Animals

FVB-background *MECP2*-duplication (Tg1) mice (Collins et al., 2004), were crossed to C57 thy1-GFP-M homozygotes obtained from The Jackson Laboratory, to generate F1C57;FVB *MECP2*-duplication;thy1-GFP-M mice and thy1-GFP-M littermate controls. Males and females were used in experiments. Animals were housed in a 12/12 h light/dark cycle (lights on from 7 A.M. to 7 P.M.). All experiments with animals were conducted in accordance with the National Institutes of Health guidelines for the care and use of laboratory animals and were approved by the institution’s Institutional Animal Care and Use Committee.

### Blinding

**Figure 1.**
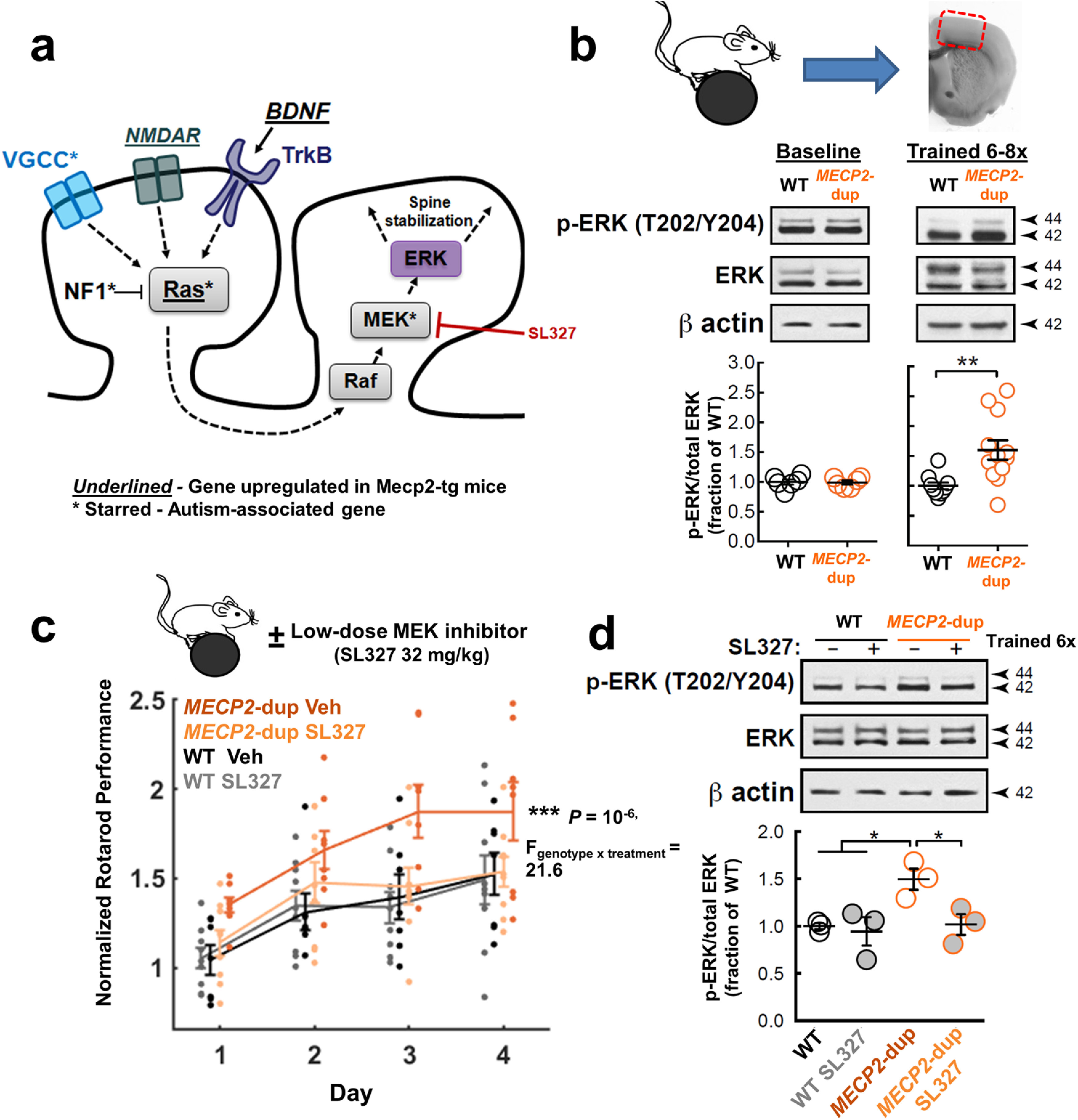
Normalization of enhanced motor learning by pharmacological inhibition of elevated Ras-ERK signaling in *MECP2*-duplication mice. ***a***, A simplified schema of the Ras-MAPK signaling pathway and how it is hypothesized to contribute to clustered-spine stabilization. Genes transcriptionally upregulated in *MECP2*-overexpressing mice are underlined. Known autism-associated genes are denoted with an asterisk. ***b***, Rotarod training (six to eight trials) induces enhanced Ras-MAPK signaling (ERK phosphorylation) in *MECP2*-duplication mice relative to WT littermates, despite equivalent baseline levels before training. Representative Western blottings and densitometric quantification of ERK activation (p-ERK T202/Y204/total ERK immunoreactivity; 42- and 44-kDa bands summed in quantification) at baseline (*n* = 6 mice/genotype; unpaired two-sided *t* test: *p *=* *0.9, *t*_(10)_ = 0.17) and after training (*n* = 10 WT and 12 *MECP2*-duplication mice; unpaired two-sided *t* test: **p =* *0.004, *t*_(20)_ = 3.2). Data are presented as fraction of the WT mean for illustration purposes. Statistical tests were performed on raw per-animal p-ERK immunoreactivity/total-ERK immunoreactivity values. Time spent on the rotarod did not explain differences between genotypes (see text). ***c***, The MEK inhibitor SL327 normalizes rotarod performance in *MECP2*-duplication mice. SL327 or vehicle was injected intraperitoneally 30 min before training on each training day. Mean ± SEM of the peak performance on each day plotted for vehicle-treated *MECP2*-duplication (dark orange, *n* = 8 mice), vehicle-treated WT (black, *n* = 7), SL327-treated *MECP2*-duplication (pale orange, *n* = 9), and SL327-treated WT (gray, *n* = 9) mice. For illustration purposes, rotarod performance for each animal was normalized to the mean first-day performance of the WT littermates in that animal’s cohort before averaging across animals, to account for systematic variability in performance across cohorts because of animal weight and age; ***p =* *10^−6^, genotype × drug interaction, *F*_genotype × treatment(1,1,29)_ = 21.6; *F*_genotype(1,29)_ = 6.4, *p *=* *0.01; *F*_treatment(1,29)_ = 4.9, *p *=* *0.027; *F*_trial(15,29)_ = 15.8, *p *=* *10^−6^; mixed effects repeated-measures ANOVA. Statistical analysis was performed on raw per-trial rotarod performance values. Litter was included as an interacting variable to control for across-litter variability in performance. ***d***, Consistent with a role for elevated ERK signaling in the mutant’s enhanced motor learning phenotype, 32 mg/kg SL327 treatment blocked the training-dependent increase in M1 ERK phosphorylation in *MECP2*-duplication mice. Representative Western blottings and densitometric quantification of ERK activation in vehicle-treated versus SL327-treated WT and *MECP2*-duplication mice (*n* = 3 mice/genotype/treatment group; *p <* *0.05, *F*_genotype(1,8)_ = 6.7, *F*_treatment(1,8)_ = 5.9, *F*_interaction(1,1,8)_ = 3.7, two-way ANOVA with Tukey’s *post hoc* correction for multiple comparisons. Normalization of data points as in ***b***. Mice in the vehicle-treated condition are also included in ***b***. Error bars represent SEM. Circles show data points from individual animals. Example full length Western blottings are shown in Extended Data Figure 1-1*a*,*b*. For blinding procedure, see Materials and Methods.

**Figure 2.**
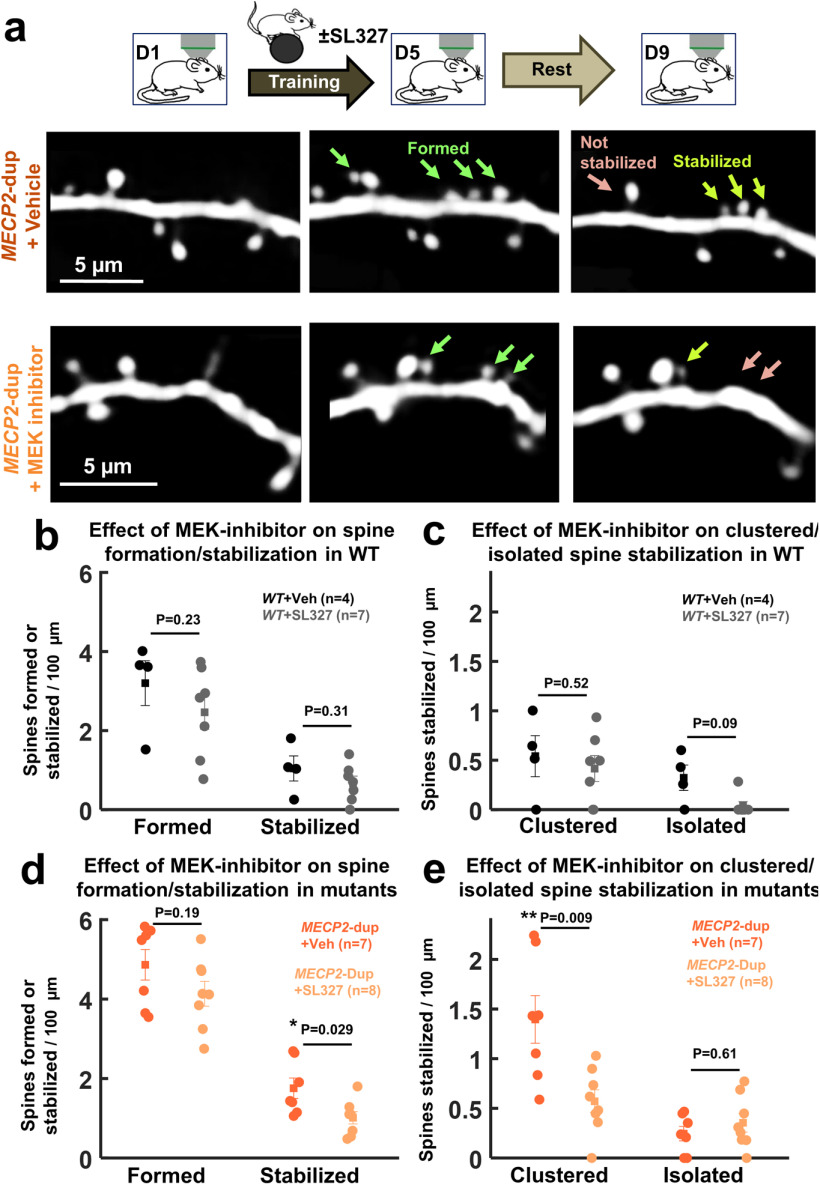
Normalization of excessive clustered spine stabilization in *MECP2*-duplication mice by pharmacological Ras-ERK inhibition. ***a***, Sample images of dendritic segments imaged before rotarod training (left), following 4 d of training (middle), and 4 d after the end of training (right) demonstrating decreased clustered spine stabilization in SL327-treated (bottom) versus vehicle-treated (top) *MECP2-*duplication mice. ***b***, Total spines formed (left bars) and spines stabilized (right bars) per 100 μm in vehicle-treated (black, *n* = 4 animals, 57 spines formed, 30 dendritic segments) and SL327-treated (gray, *n* = 7 animals, 49 spines formed, 49 dendritic segments) littermate control mice. ***c***, Clustered and isolated new-spines stabilized per 100 μm in vehicle-treated (black) and SL327-treated (gray) littermate control mice. ***d***, Total spines formed (left bars) and spines stabilized (right bars) per 100 μm in vehicle-treated (dark orange, *n* = 7 animals, 184 spines formed, 54 dendritic segments) and SL327-treated (light orange, *n* = 8 animals, 143 spines formed, 50 dendritic segments) *MECP2*-duplication mice. ***e***, Clustered and isolated spines stabilized per 100 μm in vehicle-treated (dark orange) and SL327-treated (light orange) *MECP2*-duplication mice. Note that there are significantly fewer stabilized clustered spines in SL327 treated animals; *p <* *0.05, **p <* *0.01, specific *p* values reported in the figure, Mann–Whitney *U* test.

In data reported in Figure 1, mice from each genotype were randomly assigned to the drug or vehicle condition. Material to be injected was placed into individual tubes for each animal, then mice were injected without knowledge of the test tube’s contents. Mice were trained, motor cortex was harvested, and Westerns were run and analyzed blind to condition. In Figure 2 data, imaging, rotarod training, drug/vehicle injection, and data analysis were performed blinded to experimental condition.

10.1523/ENEURO.0056-21.2021.f1-1 Extended Data Figure 1-1 Example full-length Western blottings, related to Figure 1. ***A***, Example full-length Western blottings relevant to Figure 1*B*, for p-ERK (T202/204), total ERK, and β-actin. ***B***, Example full-length Western blottings relevant to Figure 1*D*, showing immunoblots to p-ERK (T202/204), total ERK, and β-actin. Note SL-327 suppresses the level of p-ERK in mutant animals. Download Figure 1-1, TIF file.

### *In vivo* two-photon imaging

Experiments were performed as in Ash et al. (2021). At least two weeks before the first imaging session (∼12- to 14-week-old mice), a 3-mm-wide opening was drilled over motor cortex, centered at 1.6 mm lateral to bregma based on (Tennant et al., 2011), and a glass coverslip was placed over the exposed brain surface to allow chronic imaging of neuronal morphology (Mostany and Portera-Cailliau, 2008, 2011). Dendritic spines were imaged using a Zeiss *in vivo* two-photon microscope with Zeiss 20 × 1.0 NA water-immersion objective lens. High-quality craniotomies had a characteristic bright-field appearance with well-defined vasculature and pale gray matter. Under two-photon scanning fluorescent dendrites were reliably clear and visible with low laser power (<20 mW on the pia) and photomultiplier tube voltage.

Only high-quality preparations (low background noise across all time points, <5-pixel (0.25 μm) motion artifact, and dendrites well isolated from other fluorescent structures) were used in the blinded analysis. Green fluorescent protein (GFP)-labeled neurons were first imaged at low resolution (620 × 620 μm FOV, 0.6 μm/pixel in XY, 2.5-μm Z-step size) down to 600–700 μm to capture all of the cell’s dendritic processes and assay cell subtype by morphology, primary apical bifurcation depth, and soma depth (Holtmaat et al., 2006). The apical dendrites from complex-tufted neurons, the corticospinal neurons projecting to the spinal cord and thalamus in M1, were selected based on their large highly ramified dendritic trees, deep primary apical bifurcation, and thick dendrites, and re-imaged at high resolution (310 × 310 to 420 × 420 μm FOV, 0.1 μm/pixel, 1 μm Z-step size) to adequately capture individual dendritic spines. Laser power was maintained under 20 mW (average ∼10 mW) during image stack acquisition.

### Analysis of structural plasticity

Raw z stacks were denoised by a custom polynomial interpolation method (Jiang et al., 2013). Spine formation, elimination, and stabilization were quantified with a custom MATLAB user interface and ImageJ (MicroBrightField). Terminal dendrite segments which were well-visualized at all time points were chosen for analysis. Sections of dendrite occluded by other fluorescent structures or blood vessels were excluded from the analysis. Because of *in vivo* two-photon microscopy’s relatively poor resolving power in the *z*-axis, only structures protruding laterally along the X-Y plane were included in the analysis, following the standard in this field (Holtmaat et al., 2009). For a protrusion to be selected for analysis it had to project out of the dendritic shaft by at least 4 pixels (∼0.4 μm), which corresponds approximately to 2 SDs of the noise blur on either side of the dendritic shaft. Spines were initially identified at one time point, by moving up and down through individual slices in each Z stack, and the same region of dendrite was examined at other time points to identify the first (formation) and last (elimination) time that the spine was present. Custom MATLAB routines analyzed the stability/survival of each formed spine. Filopodia, which are rare at the analyzed age, were identified morphologically, based on their long length (usually >4 μm), curved shape, and lack of a distinct head (Zuo et al., 2005) and excluded from the analysis as in Yang et al. (2009). Each spine was classified as either clustered or isolated by calculating the distance to its nearest-neighbor stabilized learning-associated spine as in Fu et al. (2012) and Ash et al. (2021).

### Motor training

The Ugo Basile mouse rotarod was used for motor training. At least 2 h after imaging sessions, in the late afternoon, mice were placed on the rotarod, and the rotarod gradually accelerated from 5 to 80 rpm over 3 min. Up to five littermate mice with intermixed genotypes were trained in parallel. Single-trial rotarod performance was quantified as the time right before falling or holding on to the dowel rod for two complete rotations without regaining footing. A 5- to 10-min rest period occurred between each trial. Four trials were performed per day. For immunoblot experiments measuring M1 ERK phosphorylation with rotarod training, training-naive mice were trained six to eight times consecutively (Fig. 1*b*,*d*). For the pharmacology experiments demonstrating the effect of the specific MEK inhibitor SL327 on rotarod performance, the peak performance of each animal on each day, normalized to the vehicle-treated wild-type (WT) mean to account for across litter variability in performance, was used in the analysis (median performance yielded similar statistically significant results).

### Pharmacological Ras-MAPK inhibition

The centrally-acting selective MEK inhibitor SL327 (Atkins et al., 1998; Axonmedchem #1122) was injected intraperitoneally at 32 mg/kg (in 16 mg/ml DMSO) 30 min before rotarod training (Bureau et al., 2010). This dose was selected to be low as it is known to minimally affect motor performance in WT animals (Bureau et al., 2010).

### Immunoblots

Deeply anesthetized (isoflurane) four- to five-month-old *MECP2*-duplication mice and littermate controls were killed 30 min after training and their brains were rapidly dissected on a glass plate over ice. The motor cortex from each hemisphere was isolated from the remaining cortical tissue and lysed in ice-cold homogenization buffer (200 mm HEPES, 50 mm NaCl, 10% glycerol, 1% Triton X-100, 1 mm EDTA, 50 mm NaF, 2 mm Na_3_VO_4_, 25 mm β-glycerophosphate, and 1× EDTA-free complete ULTRA protease inhibitor cocktail tablets; Roche). Insoluble material was removed by centrifugation at 14,000 × *g* for 10 min at 4°C. Protein concentration of the resulting supernatant was determined by Bradford assay (Bio-Rad, reagent 500-0006) and lysates were then diluted in 2× Laemmli Buffer. A total of 30 μg protein/sample was resolved by SDS-PAGE (12.5% acrylamide) and gel contents were transferred to nitrocellulose membranes. Membranes were blocked 30 min in 5% milk, 0.2% Tween 20 tris-buffered saline (TBST). To assess ERK phosphorylation, membranes were first probed overnight at 4°C with phospho-specific rabbit anti-phospho-p44/42 MAPK/Erk1/2 (Cell Signaling Technology, #4370, 1:1000). Blots were then incubated with HRP-conjugated goat anti-rabbit secondary antibody (Jackson ImmunoResearch, 111-035-144, 1:5000) for 1 h at room temperature followed by incubation in Super-Signal West Femto kit substrate (Thermo Scientific, 34096) per the manufacturer’s instructions. Film was exposed to the Super-Signal-treated membranes and then developed. Membranes were then stripped (1.5% glycine and 2.9% NaCl, pH2.8), blocked with 5% milk TBST, then re-probed overnight with rabbit anti-MAPK/ERK1/2 (Cell Signaling Technologies, #9202, 1:1000) to assess total ERK protein levels. β-Actin levels were measured as an additional loading control (mouse anti-β-actin, Millipore, MAB1501, 1:5000). The goat anti-mouse secondary antibody was diluted 1:10,000 for the β-actin blots. Band density was quantified in ImageJ (NIH).

### Image presentation

Dendritic spine images are displayed as “best” projection mosaics (Holtmaat et al., 2009). Extraneous fluorescence is masked and images are slightly smoothed for illustration purposes only.

### Statistical tests

Statistical significance between unpaired normal samples was assessed by Mann–Whitney *U* test or two-way ANOVA, except where noted, using MATLAB. Enhanced rotarod performance in *MECP2*-duplication mice in Figure 1 was tested by repeated-measures ANOVA. Effect of SL327 on rotarod performance in *MECP2*-duplication mice compared with littermate controls was evaluated by determining the genotype × treatment interaction term using a mixed effects repeated-measures ANOVA in R statistics software. Statistical analysis was performed on raw per-trial rotarod performance values. Litter was included as a term to control for across-litter variability in performance; *p ≤* *0.05, **p ≤* *0.01, ***p ≤* *0.001. All results are reported as mean ± SEM, unless otherwise noted.

## Results

ERK signaling has been shown to promote cooperative plasticity between dendritic spines located at close proximity (9–10 μm) to each other along the dendrite (Harvey and Svoboda, 2007; Harvey et al., 2008). This led us naturally to hypothesize that it mediates the enhanced clustered dendritic spine stabilization previously observed in *MECP2*-duplication animals (Ash et al., 2021). The experiments described below were targeted a-priori to examine this primary hypothesis.

### Enhanced motor learning in *MECP2*-duplication mice is normalized by Ras-ERK inhibition

A simplified sketch of ERK signaling in clustered-spine plasticity is illustrated in Figure 1*a*, adapted from (Harvey et al., 2008; Ye and Carew, 2010). Several genes in the ERK pathway are associated with autism (Stornetta and Zhu, 2011; Fig. 1*a*, asterisks) and *MECP2*-duplication mice overexpress several genes in this pathway (Chahrour et al., 2008; notably, *Bdnf*, *Nmdar1*, and *Ras*; Fig. 1*a*, underlined). To directly test whether ERK signaling is increased in motor cortex of *MECP*2-duplication mice, we performed Western blot analyses on M1 protein extracts isolated from mutant mice and WT littermates at baseline and immediately following rotarod training (Fig. 1*b*; Extended Data Fig. 1-1*a*, six to eight consecutive trials in previously-untrained animals). We found that marked hyperphosphorylation of the MAP kinase ERK1/2 (T202/Y204) in *MECP2*-duplication mouse area M1 occurred with rotarod training (*p *=* *0.004, *n* = 10 WT M1 extracts from 10 mice, 12 *MECP2-*duplication M1 extracts from 12 mice, two-tailed unpaired *t* test; Fig. 1*b*). In contrast the phosphorylation state of ERK was not significantly altered in mutant M1 lysates without training (*p *=* *0.87, *n* = 6 mice per genotype, two-tailed unpaired *t* test; Fig. 1*b*), as reported previously for untrained *Mecp2*-null and *MECP2*-duplication animals (Pitcher et al., 2015). Note that the increase in ERK phosphorylation in area M1 after motor training in *MECP2*-animals could not be explained solely by increased time spent on the rotarod, as the level of phosphorylation did not correlate with the latency to fall from the rotarod (*r* = −0.17, *p* = 0.53). This suggests the induction of ERK phosphorylation by training is exuberant in the motor cortex of *MECP2*-duplication animals.

Training-induced ERK hyperphosphorylation (Fig. 1*b*) coupled with the increased clustered-spine stabilization previously observed in *MECP2*-duplication animals (Ash et al., 2021) implicated elevated ERK signaling in the enhanced motor learning phenotype of the *MECP2*-duplication syndrome mouse model (Collins et al., 2004; Harvey et al., 2008). To test this hypothesis, in a new round of experiments we administered a low dose (32 mg/kg, i.p.) of the specific centrally-acting MEK inhibitor SL327 or DMSO vehicle 30 min before rotarod training on each day (Bureau et al., 2010), and trained the animals for four consecutive trials daily for four days in a row (Collins et al., 2004; Ash et al., 2021). This dose was chosen to ensure effects on locomotor function in WT are minimal (Antoine et al., 2013; but see Bureau et al., 2010). Accordingly, the performance of WT mice was not affected, whereas SL327 reversed the enhanced motor learning in mutants (*p *=* *10^−6^, genotype × drug interaction, *F*_genotype × treatment_ = 21.6, *n* = 7–9 mice per group, mixed effects repeated-measures ANOVA; Fig. 1*c*). Furthermore, M1 ERK phosphorylation was normalized in SL327-treated mutants versus vehicle-treated mutants (Fig. 1*d*; Extended Data Fig. 1-1*b*, *p *<* *0.05, *n* = 3 mice/group, two-way ANOVA with Tukey’s *post hoc* correction for multiple comparisons), confirming the effectiveness of the inhibitor in reducing ERK phosphorylation levels.

### Enhanced clustered spine stabilization in *MECP2*-duplication mice is normalized by Ras-ERK inhibition

Lastly, we measured the effect of MEK inhibition on clustered spine stabilization in *MECP2-*duplication mice and littermate controls crossed to the thy1-GFP M line, which sparsely expresses GFP in layer 5 pyramidal neurons (Feng et al., 2000). As in Ash et al. (2021), chronic cranial windows were implanted over primary motor cortex (M1) in *MECP2*-duplication mice and littermate controls, and apical dendrites from GFP-expressing L5 pyramidal neurons were imaged with *in vivo* two-photon microscopy (Holtmaat et al., 2009). Spine analysis was performed on terminal dendritic branches of the apical tuft of these neurons. We first identified baseline spines, then animals were trained on the rotarod task (four trials per day) for 4 d (Fig. 2*A*). Animals were randomized to receive SL327 32 mg/kg or vehicle (DMSO) injected intraperitoneally 30 min before training across the four rotarod training days. On the fifth day, dendrites were imaged again to identify new spines formed. Following 4 d of rest, dendrites were once again imaged to identify the new spines that stabilized. The follow-up imaging time point was chosen in line with prior studies showing that the vast majority of newly formed dendritic spines which persist for at least 4 d form an electron-microscopy-verified synapse (Knott et al., 2006). Each stabilized spine was classified as clustered or isolated based on its proximity to another newly-formed spine on the same dendrite (clustered: <9 μm interspine distance; isolated: ≥9-μm interspine distance, following Ash et al., 2021).

As observed previously (Ash et al., 2021), clustered spine stabilization was increased in vehicle-treated *MECP2*-duplication mice compared with controls (WT 1.1 ± 0.2 stabilized per 100 μm, n =4 animals, 30 dendritic segments; *MECP2*-duplication: 2.2 ± 0.4 stabilized per 100 μm, *n* = 7 animals, 54 dendritic segments, *p *=* *0.04, Mann–Whitney *U* test; two-way ANOVA effect of genotype: *p* = 0.03, *F*_(1,22)_ = 5.5). Overall spine formation/stabilization as well as clustered and isolated spine stabilization were not significantly affected by MEK inhibition in control mice (all *p* values > 0.09, Mann–Whitney *U* test, *n* = 4 vehicle-treated WT mice, 7 SL327-treated WT mice; Fig. 2*b*,*c*), as expected given the low dose of SL327 (Antoine et al., 2013). In contrast, in *MECP2*-duplication animals overall spine stabilization was significantly reduced by SL327 (effect on stabilization: *p *=* *0.029, Mann–Whitney *U* test; two-way ANOVA effect of SL327: *p* = 0.02, *F*_(1,22)_ = 6.2; interaction: *p* = 0.4; *n* = 7 vehicle-treated *MECP2*-duplication mice, *n* = 8 SL327-treated *MECP2*-duplication mice; Fig. 2*d*), back to levels similar to that of WT controls (SL327-treated mutant spine stabilization rate: 1.0 ± 0.2 per 100 μm; vehicle-treated WT spine stabilization rate: 1.0 ± 0.3 per 100 μm, vehicle-treated mutant spine stabilization rate: 1.7 ± 0.2). Separating stabilized spines into clustered and non-clustered subgroups revealed that this SL327-induced decrease in spine stabilization was mediated entirely by a reduction in the stabilization of clustered spines (*p *=* *0.009, Mann–Whitney *U* test, Cohen’s *d *=* *1.65; two-way ANOVA effect of SL327: *p* = 0.01, *F*_(1,22)_ = 7.7, effect of SL327; effect of genotype: *p* = 0.01, *F*_(1,22)_ = 7.7; Interaction: *p* = 0.06, *F*_(1,22)_ = 3.7; *n* = 7 vehicle-treated *MECP2*-duplication mice, *n* = 8 SL327-treated *MECP2*-duplication mice; Fig. 2*e*). SL327 had a negligible effect on the stabilization of isolated new-spines in mutants (*p *=* *0.61, Mann–Whitney *U* test, Cohen’s *d *=* *0.3) and also did not affect significantly overall spine formation (effect on mutant formation: *p *=* *0.19, Mann–Whitney *U* test; two-way ANOVA effect of SL327: *p* = 0.09; effect of genotype: *p* = 0.0006, *F*_(1,22)_ = 15.9; interaction: *p* = 0.9; Fig. 2*d*). Interestingly, there was a trend toward decreased stabilization of *isolated* spines in SL327-treated WT mice not seen in mutants (genotype × SL327 interaction: *p* = 0.03, *F*_(1,22)_ = 5.2), although this trend did not survive pairwise comparison (*p* > 0.1, Mann–Whitney *U* test). These observations suggest that elevated ERK signaling contributes to the structural stabilization of training-associated clustered synapses in the *MECP2*-duplication mouse.

## Discussion

In summary, we found that the ERK pathway, a major regulator of clustered spine stabilization (Harvey et al., 2008; Frank et al., 2018), is hyperactive following training in *MECP2*-duplication mouse motor cortex, and both increased spine-cluster stabilization and enhanced motor learning in *MECP2*-duplication mice can be reversed by ERK-specific pharmacologic inhibition.

Our results build on a growing evidence base implicating Ras-ERK/MAPK signaling in ASD (Vithayathil et al., 2018). Mutations in Ras-MAPK pathway genes including NF1, Ras, MEK, and RSK, together referred to as Rasopathies, cause syndromic autism (Stornetta and Zhu, 2011). Pathway analyses of large scale genome sequencing data confirm that Ras-MAPK pathway mutations are enriched in idiopathic autism (Wen et al., 2016; Mitra et al., 2017), and upregulations in Ras-MAPK pathway activity correlate with symptom severity in autism patients (Rosina et al., 2019). Multiple animal models of autism demonstrate abnormalities in Ras-MAPK signaling including Rett syndrome (Mellios et al., 2018), BTBR strain (Seese et al., 2014; Cheng et al., 2017), 16p11del (Pucilowska et al., 2015), fragile X syndrome (Soong et al., 2008), neurofibromatosis type 1 (Costa et al., 2002), Noonan syndrome (Lee et al., 2014), Costello syndrome (Schreiber et al., 2017), cardio-cutaneo-facial syndrome (Anastasaki et al., 2009), and SynGAP1 syndrome (Rumbaugh et al., 2006). Autism-associated phenotypes have been shown to be rescued by inhibition of Ras-ERK signaling in many of these animal models (Wang et al., 2012; Pucilowska et al., 2018; Moreau et al., 2020; Mullins, 2020). Our results provide the first evidence that Ras-ERK signaling is upregulated following training in the mouse model for *MECP2*-duplication syndrome, and that its abnormally enhanced motor learning phenotype can be rescued by inhibiting ERK signaling (Fig. 1).

Essentially all autism models that have been formally tested exhibit changes in synaptic plasticity and learning (Bourgeron, 2015), including *MECP2*-duplication mice (Collins et al., 2004; Na et al., 2012; Jiang et al., 2013; Ash et al., 2018). In prior work, it was shown that clustered spine consolidation is abnormally enhanced in *MECP2*-duplication animals (Ash et al., 2021). Here, we show that *MECP2*-duplication clustered spine consolidation returned to WT-like levels after normalizing ERK signaling with the specific pharmacological inhibitor SL327 (Fig. 2). Furthermore, this occurred at a dose that did not alter the rate of new spine formation or the rate of new isolated spine consolidation after training, so the observed effect appears to be specific to co-operative spine consolidation. These observations forge a link between increased ERK signaling, increased clustered spine consolidation, and enhanced learning phenotype in the *MECP2*-duplication mouse model of syndromic autism.

Our results align with converging evidence on the role of ERK signaling in dendritic spine clustering and autism pathophysiology. Multiple links in the Ras-ERK pathway including BDNF, TrkB, Ras, and ERK are required for clustered spine consolidation *ex vivo* (Harvey et al., 2008; Niculescu et al., 2018), and CCR5-mutant animals engineered to have hyperactive Ras-ERK signaling exhibit increased clustered spine plasticity (Frank et al., 2018). Our contribution adds to this work, showing that the excessive clustered spine stabilization previously observed in the *MECP2*-duplication syndrome mouse model (Ash et al., 2021) can be normalized *in vivo* with a pharmacological inhibitor of ERK signaling.

One remaining puzzle is the fact that excessive clustered spine stabilization in *MECP2*-duplication animals occurs both with and without training (Ash et al., 2021), whereas excessive ERK phosphorylation occurred with training but not without training (Fig. 1*b*; Pitcher et al., 2015). It is possible that a subtle elevation in Ras-MAPK signaling (ERK phosphorylation) may be present without training, but not be enough to be detected by Western blot analysis (White and Wolf-Yadlin, 2016), as any difference at baseline would likely be small given our data (Fig. 1*b*). Other nonexclusive possibilities include that downstream effectors of p-ERK are nonlinearly affected by Ras-ERK activity in *MECP2*-duplication mice, and/or they can still be activated through ERK-independent mechanisms without training. Finally, Ras-ERK inhibition may modulate motor learning and spine consolidation through mechanisms other than Ras’s role in dendritic spines. Ras-ERK modulates learning and plasticity in the brain through multiple presynaptic and postsynaptic mechanisms in both excitatory and inhibitory neurons (Ryu and Lee, 2016). It is not inconceivable, for example, that MEK inhibition could downregulate spontaneous activity or alter modulatory neurotransmitter release (Ding et al., 2011; Borges et al., 2017), which would in turn indirectly normalize enhanced learning and clustered spine consolidation.

Importantly, we note that we have not conclusively shown that clustered spine stabilization drives enhanced learning, although others previously showed a correlation between spine clustering and learning across animals (Frank et al., 2018; Ash et al., 2021). The fact that the MEK inhibitor normalized motor performance in *MECP2*-duplication mice even on the first day of training (Fig. 1*c*), at which point clustered spine stabilization is unlikely to play a significant role, suggests that excessive Ras-MAPK signaling boosts learning in mutants through multiple mechanisms. Verifying a causal link between clustered spine consolidation and learning is an important goal of future work. Finally, we note that in our preparation 32 mg/kg SL327 did not significantly affect WT rotarod performance or M1 ERK phosphorylation, which aligns with (Antoine et al., 2013) but disagrees somewhat with (Bureau et al., 2010), who reported that 30 mg/kg SL327 was sufficient to decrease rotarod performance in WT mice and decrease ERK phosphorylation in the striatum. It is possible that subtle differences in the rotarod training parameters (four trials per day for 4 d, accelerating from 5 to 80 rpm in 3 min in our paradigm, vs 10 trials per day for 4 d, from 4 to 40 rpm in 5 min in their paradigm) and probing for ERK in different brain areas could explain these discrepancies, but this requires follow-up experimental assessment to determine under which conditions SL327 does or does not affect WT learning and memory.

Although enhanced motor learning is not observed in human patients (Ramocki et al., 2010), it is possible that the structural abnormality we describe in mice is also operating in the humans, but manifests with a different developmental trajectory of motor deterioration because of species-specific differences in motor system development and function (Collins et al., 2004; Ramocki et al., 2009). We note again here that several autism mouse models including *MECP2*-duplication, neuroligin-3, 15q duplication, PTEN, CNTNAP2, and CCR5 have enhanced motor learning and plasticity (Etherton et al., 2011; Piochon et al., 2014; Rothwell et al., 2014; Frank et al., 2018), while several other mouse models including Rett syndrome, fragile X, and Angelman (15q deletion), have impaired motor learning and plasticity (Li et al., 2002; Asaka et al., 2006; Van Woerden et al., 2007; Kondo et al., 2008; Padmashri et al., 2013). In particular, opposite to our findings in the *MECP2*-duplication mice, it was recently reported that fragile X mice have impaired motor learning and decreased clustered spine stabilization (Bhattacharya et al., 2012; Padmashri et al., 2013; Reiner and Dunaevsky, 2015). Building on other studies positing an axis of synaptic pathophysiology in syndromic autism (Auerbach et al., 2011), these findings together suggest that certain forms of syndromic autism defined by enhanced plasticity and prominent behavioral inflexibility could be particularly amenable to Ras-MAPK modulating agents.

Our results provide *in vivo* evidence that ERK signaling is involved in learning-associated synaptic stabilization and synaptic clustering and suggest that a training-dependent increase in M1 ERK signaling facilitates procedural motor memory consolidation in *MECP2*-duplication animals. Our findings provide additional support to hypotheses that learning phenotypes in autism arise from disrupted Ras-ERK signaling and synaptic plasticity (Bourgeron, 2015; Vithayathil et al., 2018). In the future, it will be fruitful to study the regulation of upstream and downstream mediators of ERK signaling in *MECP2*-duplication mice and explore how excessive ERK signaling contributes to excessive clustered spine stabilization in this mouse model (Alonso et al., 2004; Im et al., 2010; Ye and Carew, 2010; Stornetta and Zhu, 2011; Ebert and Greenberg, 2013; Niculescu et al., 2018). In the future, modulation of structural plasticity through Ras-MAPK inhibitors could provide a potential therapeutic avenue for behavioral inflexibility and other phenotypes in the *MECP2*-duplication syndrome (Sandweiss et al., 2020).

## Synthesis

Reviewing Editor: Christian Hansel, University of Chicago Decisions are customarily a result of the Reviewing Editor and the peer reviewers coming together and discussing their recommendations until a consensus is reached. When revisions are invited, a fact-based synthesis statement explaining their decision and outlining what is needed to prepare a revision will be listed below. The following reviewer(s) agreed to reveal their identity: Peyman Golshani, Stéphane Baudouin.

The manuscript shows convincing evidence of a link between dysregulation of ERK signaling, synaptic structural plasticity, and motor deficits in mice with duplication of MeCP2. This work adds to existing scientific evidence showing, in preclinical models, and involvement of Ras-ERK signaling in the pathophysiology of neurodevelopmental disorders, and autism spectrum disorders in particular.  These results also replicate results previously obtain in the MeCP2 duplication mouse model (Ash et al 2020) providing important confirmation of published results.

The authors find hyperphosphorylation of ERK 1/2 in the MECP2-duplication mouse only after rotarod training. The MECP2 duplication mice have enhanced motor learning and treatment with MEK inhibitor SL327 blocked this enhancement and blocked clustered stabilization of L5 apical dendritic spines in motor cortex. Overall this is an important paper with exciting findings that have potential relevance to understanding aberrant plasticity in MECP2 duplication and Rett’s. The experiments are well-designed, the analysis is robust and the effect sizes large.

Detailed critique:  Abstract/Introduction:  I think it would be important for the readers that the authors clarify the relationship between the MeCP2 duplication mouse model and ASD, and in particular address the following points:  1- In the abstract, the authors use the term syndromic autism. This term refers to a very specific notion in ASD, relating to two categories of ASD. The authors should clarify this notion in the introduction  2- The MeCP2 duplication mouse model is not well-studied compared to the MeCP2 deletion model. I would therefore encourage the authors to provide additional background to better describe the model, particularly to clarify the existence of the two models and briefly highlight their differences. Such an introduction could also serve to better present the link between MeCP2, Rett syndrome, and autism spectrum disorders.  3- The authors relate the phenotype of the MeCP2 duplication mouse model to ASD and, in the current manuscript, this behavior is enhanced learning. I do not think that enhanced learning is a characteristic feature of ASD (see my related comment in the discussion). The authors should provide a more in-depth justification in relating their results to ASD and allude to it in the introduction to avoid misleading the readers.   Comments related to Figure 1:  - The results are in 1B, 1C, and 1D are presented as a fraction of WT and there are multiple data points for WT. I find the nomenclature unclear; it would be better to more clearly state what the ratio is exactly. It would also be important to state which data were used for the statistical analysis.  - For 1C specifically, it would be good if the authors could present the results in more detail, as they are presented in Ash et al 2020 for example.  - The authors showed an absence of the effect of SL327 on the level of phosphorylation of ERK in WT animals. The argument used by the authors to justify such absence of an effect is that the dose used, 32mg/kg, is too low to affect wild-type animals, referencing Antoine et al. 2013. Bureau et al 2010, also cited in the article, showed that treatment with 30mg/kg SL327 decreases ERK phosphorylation in WT. It would be good if the authors fully acknowledge the existence of these results and discuss the discrepancies. A possible explanation for the discrepancy is the use of different brain regions (striatum versus M1). Also, ERK phosphorylation is increased in wild-type animals following rotarod training (Bureau et al 2020), unlike M1. The authors could try to determine, experimentally, if the difference in brain regions is the reason for the discrepancy.  - Similarly, Bureau et al 2020 showed that treatment with 30mg/kg of SL327 impaired motor learning in wild-type mice. This effect is not replicated by the authors but also not mentioned. As above, it would be important to better acknowledge existing results and discuss potential reasons for the discrepancy.  - It is unclear to me if SL327 is injected every day before rotarod training, please clarify.  Note that I do not think that an absence of replication necessarily invalids the present results.   Comments related to Figure 2:  - The results are presented in a very synthetic form, using bar graph representations. For reasons of clarity in the results but also consistency in the presentation between figures, it would be better to represent individual data points.  - As noted by the authors, results in Figure 1C showed a potential trend for an effect of SL327 on the stability of isolated spines. The number of animals used in this study is rather small compared to Ash et al 2020, which is directly comparable, and it remains possible that such a low number affects the results of the statistical analysis. Such a result could prove important when stated that 30mg/kg of SL327 does not affect wild-type animals. If possible, it would be good to add data points to more firmly conclude on an absence of effect of SL327 in wild-type mice.   Discussion:  The authors state that “These observations forge a link between increased ERK signaling, increased clustered spine consolidation, and enhanced learning phenotype in the MECP2-duplication mouse model of syndromic autism”. The authors also state their “findings provide additional support to hypotheses that learning phenotypes in autism arise from disrupted Ras-ERK signaling and synaptic plasticity”.  Enhanced learning is not a feature particularly associated with ASD and so it would be interesting to develop on the nature of the link between enhanced learning and phenotypes associated with ASD. In particular, it would be interesting to know to which category or subtype of ASD could benefit from treatment with ERK inhibitors.

Minor criticisms:   1. The authors should include the individual data points on top of the bar graphs for Figure 2B-E.  2. Do the authors have any information on the involvement of different spine types in the MECP2 duplication phenotype?

## Author Response

Dear Dr. Hansel,

We are glad that our work was well-received by the reviewers, and we were highly motivated to address

their issues and suggestions. Please forgive the delayed revision response, which was necessary due to

the first author’s clinical duties. The reviewers make several constructive and addressable issues related

to readability and data presentation. We are sincerely appreciative of these comments, and in

addressing them we feel that we have made the manuscript a more rigorous and readable scientific

work.

Here we reproduce the reviewers’ comments and our revisions point-by-point:

The manuscript shows convincing evidence of a link between dysregulation of ERK signaling, synaptic

structural plasticity, and motor deficits in mice with duplication of MeCP2. This work adds to existing

scientific evidence showing, in preclinical models, and involvement of Ras-ERK signaling in the

pathophysiology of neurodevelopmental disorders, and autism spectrum disorders in particular. These

results also replicate results previously obtain in the MeCP2 duplication mouse model (Ash et al 2020)

providing important confirmation of published results.

The authors find hyperphosphorylation of ERK 1/2 in the MECP2-duplication mouse only after rotarod

training. The MECP2 duplication mice have enhanced motor learning and treatment with MEK inhibitor

SL327 blocked this enhancement and blocked clustered stabilization of L5 apical dendritic spines in motor

cortex. Overall this is an important paper with exciting findings that have potential relevance to

understanding aberrant plasticity in MECP2 duplication and Rett’s. The experiments are well-designed,

the analysis is robust and the effect sizes large.

Detailed critique:

Abstract/Introduction:

I think it would be important for the readers that the authors clarify the relationship between the MeCP2

duplication mouse model and ASD, and in particular address the following points:

-In the abstract, the authors use the term syndromic autism. This term refers to a very specific

notion in ASD, relating to two categories of ASD. The authors should clarify this notion in the

introduction

--The MeCP2 duplication mouse model is not well-studied compared to the MeCP2 deletion

model. I would therefore encourage the authors to provide additional background to better

describe the model, particularly to clarify the existence of the two models and briefly highlight

their differences. Such an introduction could also serve to better present the link between

MeCP2, Rett syndrome, and autism spectrum disorders.

--The authors relate the phenotype of the MeCP2 duplication mouse model to ASD and, in the

current manuscript, this behavior is enhanced learning. I do not think that enhanced learning is a

characteristic feature of ASD (see my related comment in the discussion). The authors should

provide a more in-depth justification in relating their results to ASD and allude to it in the

introduction to avoid misleading the readers. 2

We are very thankful to the reviewer for these constructive suggestions to improve the

readability and rigor of the abstract and introduction. We have modified these sections based

on the feedback and are happy with the new version.

Comments related to Figure 1:

The results are in 1B, 1C, and 1D are presented as a fraction of WT and there are multiple data

points for WT. I find the nomenclature unclear; it would be better to more clearly state what the

ratio is exactly. It would also be important to state which data were used for the statistical

analysis.

Each data point in Fig. 1b and 1d depicts a single animal's phospho-ERK T202/Y204

immunoreactivity / total ERK immunoreactivity, divided by the WT mean for illustration

purposes. Statistical tests were performed on raw per-animal p-ERK immunoreactivity / total-ERK immunoreactivity values. In Fig. 1c, rotarod performance for each animal was normalized

to the mean first-day performance of the WT littermates in that animal’s cohort, to account

for systematic variability in performance across cohorts due to animal weight and age. This

was done for illustration purposes only, and statistical analysis was performed on raw per-trial rotarod performance values (with litter as an interacting random variable).

We have updated the figure legend in Fig. 1 as suggested by the reviewer to assist in

readability and interpretability of the results.

For 1C specifically, it would be good if the authors could present the results in more detail, as

they are presented in Ash et al 2020 for example.

We appreciate this suggestion. We added individual data points in Fig. 1c, which better

visualizes the robustness of the results.

- The authors showed an absence of the effect of SL327 on the level of phosphorylation of ERK in

WT animals. The argument used by the authors to justify such absence of an effect is that the

dose used, 32mg/kg, is too low to affect wild-type animals, referencing Antoine et al. 2013.

Bureau et al 2010, also cited in the article, showed that treatment with 30mg/kg SL327

decreases ERK phosphorylation in WT. It would be good if the authors fully acknowledge the

existence of these results and discuss the discrepancies. A possible explanation for the

discrepancy is the use of different brain regions (striatum versus M1). Also, ERK phosphorylation

is increased in wild-type animals following rotarod training (Bureau et al 2020), unlike M1. The

authors could try to determine, experimentally, if the difference in brain regions is the reason for

the discrepancy. Similarly, Bureau et al 2020 showed that treatment with 30mg/kg of SL327

impaired motor learning in wild-type mice. This effect is not replicated by the authors but also

not mentioned. As above, it would be important to better acknowledge existing results and

discuss potential reasons for the discrepancy... Note that I do not think that an absence of

replication necessarily invalids the present results.

We are thankful for these suggestions and agree with the reviewer’s contentions. We have

updated the results and discussion to point out these discrepancies between our results and

that of Bureau et al 2010, and describing key methodological differences between the studies.

It is unclear to me if SL327 is injected every day before rotarod training, please clarify.

We apologize that this was not clear. Yes, SL327 was injected 30 minutes prior to training on

all 4 training days. We updated the results and figure legend to make this more clear.3

Comments related to Figure 2:

- The results are presented in a very synthetic form, using bar graph representations. For reasons of

clarity in the results but also consistency in the presentation between figures, it would be better to

represent individual data points.

We agree that this helps visualize the robustness of the results. We updated the plots in Fig. 2 to

include individual data points and are happy with the result.

- As noted by the authors, results in Figure 2C showed a potential trend for an effect of SL327 on the

stability of isolated spines. The number of animals used in this study is rather small compared to Ash et al

2020, which is directly comparable, and it remains possible that such a low number affects the results of

the statistical analysis. Such a result could prove important when stated that 30mg/kg of SL327 does not

affect wild-type animals. If possible, it would be good to add data points to more firmly conclude on an

absence of effect of SL327 in wild-type mice.

We agree that this is potentially an exciting finding worthy of follow-up and are glad that the reviewer

noticed it, and we also recognize that the results are not adequately powered to make a strong claim.

We are working to optimize both our method of plasticity induction and readout, as well as our

method for measuring and modulating Ras-ERK activity, as part of future work to explore these and

related questions, but this is part of a larger project and we feel is beyond the scope of the current

manuscript.

Discussion:

The authors state that “These observations forge a link between increased ERK signaling, increased

clustered spine consolidation, and enhanced learning phenotype in the MECP2-duplication mouse model

of syndromic autism”. The authors also state their “findings provide additional support to hypotheses

that learning phenotypes in autism arise from disrupted Ras-ERK signaling and synaptic plasticity”.

Enhanced learning is not a feature particularly associated with ASD and so it would be interesting to

develop on the nature of the link between enhanced learning and phenotypes associated with ASD. In

particular, it would be interesting to know to which category or subtype of ASD could benefit from

treatment with ERK inhibitors.

We have thought a good amount about these issues and are glad that the reviewer called attention to

them. We decided to include a slightly expanded discussion on these themes while recognizing that

they are somewhat speculative.

Minor criticisms:

1- The authors should include the individual data points on top of the bar graphs for Figure 2B-E.

Done

2- Do the authors have any information on the involvement of different spine types in the MECP2

duplication phenotype?

We first note here that in Jiang et al J Neuro 2013 subtle differences in the densities and

morphologies of different spine types were observed in somatosensory cortex of MECP2-

duplication mice. In pilot studies we attempted to separate spines by subtypes (mushroom,

stubby, thin/filopodia). We found that the filopodia are never stabilized, so we excluded them 4

from the analysis (as stated in the methods). In our hands we did not see prominent

differences in spine formation and stabilization between mushroom and stubby spines, so we

pooled these spine types to simplify the spine counting step (which is a very time-consuming

part of the analysis)
