# Supplementary figures and images for: Inhibition of Elevated Ras-MAPK Signaling Normalizes Enhanced Motor Learning and Excessive Clustered Dendritic Spine Stabilization in the MECP2-Duplication Syndrome Mouse Model of Autism
Source: eNeuro. 2021 Jul 3;8(4):ENEURO.0056-21.2021. doi: 10.1523/ENEURO.0056-21.2021 (PMC8260274; doi:10.1523/ENEURO.0056-21.2021)

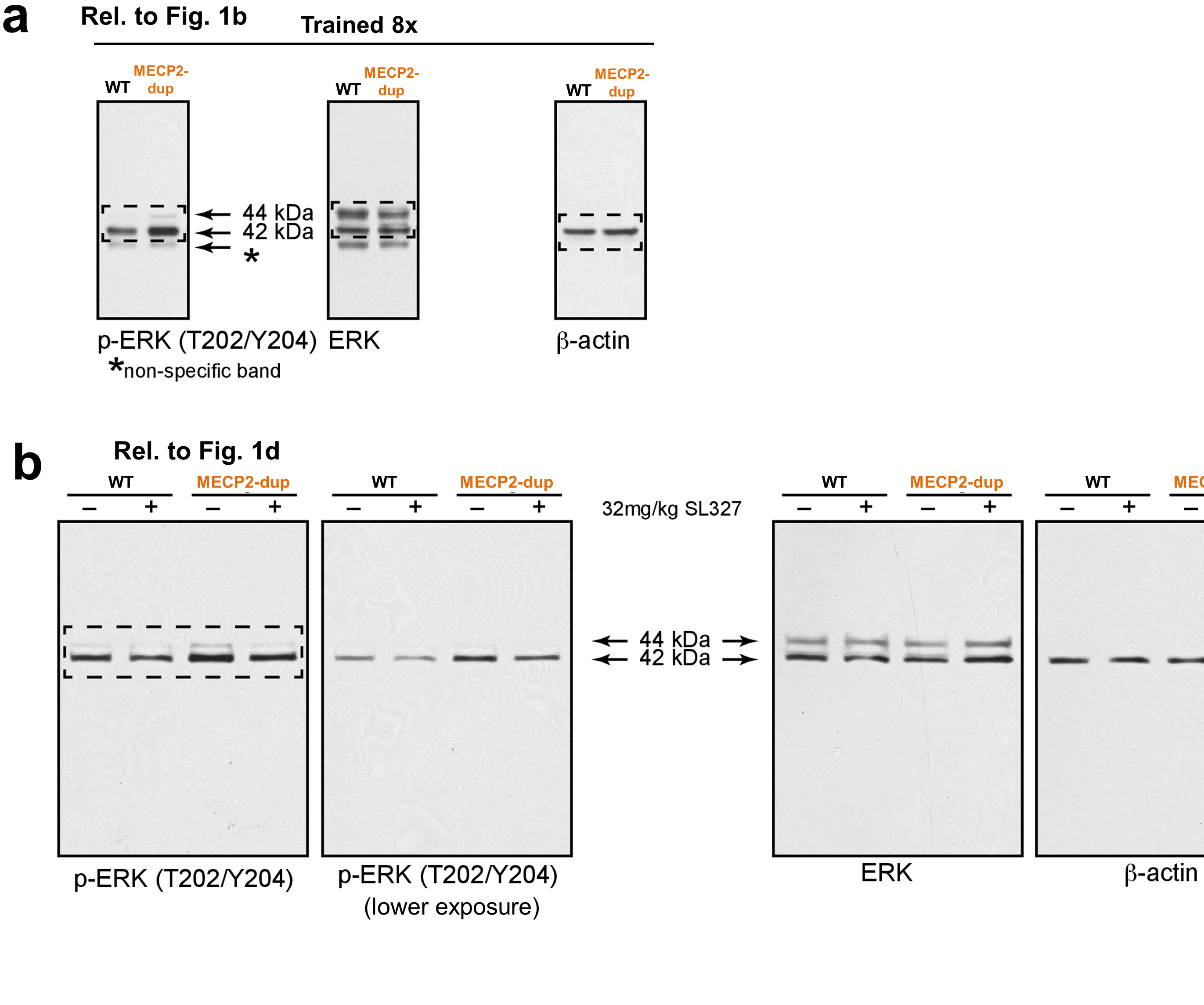

Supplement: Extended Data Figure 1-1 — Example full-length Western blottings, related to Figure 1. A, Example full-length Western blottings relevant to Figure 1B, for p-ERK (T202/204), total ERK, and β-actin. B, Example full-length Western blottings relevant to Figure 1D, showing immunoblots to p-ERK (T202/204), total ERK, and β-actin. Note SL-327 suppresses the level of p-ERK in mutant animals. Download Figure 1-1, TIF file. [file enu-eN-NWR-0056-21-s01.tif]
